# Small journal vs. high impact journal: Dilemma of a new author

**DOI:** 10.4103/0253-7613.55201

**Published:** 2009-06

**Authors:** D. K. Sahu

**Affiliations:** Indian Journal of Medical Sciences, Mumbai, India. E-mail: dksahu@medknow.com

Where to publish, asks the new author. An author faces a dilemma as s/he weighs the advantages of lower rejection/faster turnaround time of a small journal *versus* the higher rejection rate and may be long waiting period (for publication after acceptance) by a ‘high impact’ journal. S/he looks for two important parameters in a peer-reviewed journal before submitting a manuscript *viz*. how quickly can one get the paper published and what impact the published paper will have thanks to the journal *per se*. Someone who needs an ‘immediate’ acceptance letter for a fast approaching date for review, promotion or grant application would opt for a journal which can accept a paper early (i.e. has a fast turnaround time) or easily (i.e. has a low rejection rate). Some promotional or grant reviews give more preference to journals indexed with PubMed or Science Citation Index (SCI). Many job applications in research institutions and grant applications request the applicant to list their publications based on the Impact Factor (IF) of the journal. It is considered that papers published in journals with high IF or indexed with PubMed /SCI are likely to have a higher research impact compared to papers published in ‘non-indexed’ or ‘low-IF’ journals. Hence, a prospective author gives preference to journals with this indexing or recognition.

Apart from looking for the turnaround time and rejection rates, an author today looks for ease and convenience of the submission process of a given journal. About a decade ago, majority of the journals requested authors to submit at least three sets of neatly typed manuscripts double spaced, with 2.5 inches of margin on all the sides, printed on a good quality white paper. One had to submit three sets of glossy photographs, if the paper had any figures, along with the original submission. This wasn't all the hard work one had to do; the revision process was equally tedious. Authors were required to submit one or sometimes three sets of revised manuscripts along with one copy with underlined or colored text to highlight the changes done. Imagine if a manuscript had to undergone three or four revisions. With advancing technology, scholarly journals today are more author-friendly than before. Majority of the journals encourage electronic submission of manuscripts either through email or a web-based manuscript submission system. The process of submission is so simple for the new age technology savvy authors that simultaneous submissions of a single manuscript to more than one journal are often seen! Editors like me who have worked on more than one journal at a time have, on many occasions, reviewed a manuscript rejected just a few minutes back by another journal. While most submission systems are quite advanced and user-friendly there are a few issues which authors have to face. Most such systems are developed for publishers who can automatize most of the publishing tasks till the print or web publication of the journal. To capture every possible data, in ready to use XML, authors spend a little more time than they wish to. The web-based submission system may seem a bit impersonal for some authors because of the automated template based electronic mails, some of which even address them as ‘Dear Author(s)’. The local environment on the computer (such as web browser, internet connection speed, network securities, etc.) and differences in various submission systems also affect user-friendliness for authors. Overall, however, these submission systems have expanded the umbrella of journals where one can submit manuscripts and also helped journals reduce the turnaround time for submitted manuscripts.

The Indian Journal of Pharmacology (IJP) has been using a web-based manuscript submission system (www.journalonweb.com/ijp) for over five years now. The system allows authors to submit a manuscript within minutes by attaching two document files of a manuscript. The authors can track the progress of the manuscript during the peer-review process. A reference checking facility helps the authors check correctness of references cited in the manuscript. A new feature added recently alerts authors by a mobile message when a manuscript is sent for revision or proof reading. For editors, the system allows to check for possible plagiarism or duplicate submission. Editors can send a manuscript to any number of reviewers not limiting to any particular country or place. The purpose of using such a system is to facilitate faster peer review of submitted manuscripts.

When the author hopes for high impact of his or her work through publication in a prestigious high impact journal, s/he is actually looking for maximum possible readers. The author expects the journal's visibility and credibility to get readers who would use his/her research and build upon it. Readers cite his/her work in their future publication. The author anticipates gain in both research and citation impact through publishing in such a journal. An alternative to publishing in a high impact journal is now available with the advent of open access (OA) journals. Traditional subscription-based journals face competition from the new OA journals which offer immediate free access to published research papers through a web-based medium. Some OA journals, however, charge the authors a fee for submission or publication of a manuscript. Majority of the OA journals, fortunately, do not have such ‘author-side’ fee. Articles made available freely on the internet through an OA journal or interoperable OA repository have shown to have higher visibility and research and citation impact than those published in journals with a subscription barrier. Some subscription-based ‘hybrid’ journals also offer authors the option of paying a fee to make their paper available for free to the readers. The purpose of making research available for a wider audience through OA is to gain a higher impact which otherwise is lost due to the access barriers of subscription based journals.

**Figure 1:**
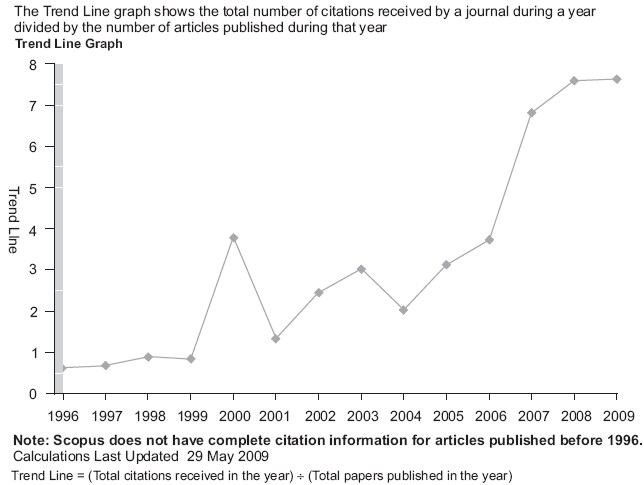
Citation trends for the Indian Journal of Pharmacology from 1996 to 2009 (Source: SCOPUS)

The IJP offers a platform for wider visibility of published research through its OA website (www.ijp-online.com). Like most OA journals from India and other developing countries, IJP does not collect any fee from authors for submission, processing or publication of manuscripts. All articles including review articles published in IJP are made available online on its website and on Bioline International's website (www.bioline.org.br) even before the print publication. The effect of this wider visibility has been seen on the citations received to the journal's articles [Figure 1] which also help in the indexing of the IJP with the SCI. Apart from providing free access (both in HTML and PDF), the IJP's website has many features which would interest most readers. The site provides reference linking, citation tracking, citation alerts, facility to add comments, statistics on downloads of articles, mapping of authors on Google Map, social networking and bookmarking tools and many more. The journal also permits and encourages authors to use full text PDF files of articles for archiving on an institutional or a subject based interoperable repository. The purpose of this OA policy of IJP is to ensure highest possible impact for papers published in IJP. It is expected that the authors would appreciate these efforts made by IJP and ensure that their valuable research is submitted here as the first priority.

